# Dynamic category-sensitive hypergraph inferring and homo-heterogeneous neighbor feature learning for drug-related microbe prediction

**DOI:** 10.1093/bioinformatics/btae562

**Published:** 2024-09-18

**Authors:** Ping Xuan, Zelong Xu, Hui Cui, Jing Gu, Cheng Liu, Tiangang Zhang, Peiliang Wu

**Affiliations:** School of Information Science and Engineering, Yanshan University, Qinhuangdao 066004, China; Department of Computer Science and Technology, Shantou University, Shantou 515063, China; School of Information Science and Engineering, Yanshan University, Qinhuangdao 066004, China; Department of Computer Science and Information Technology, La Trobe University, Melbourne, VIC 3083, Australia; Australian Centre for AI in Medical Innovation, La Trobe University, Melbourne 3083, Australia; School of Computer Science and Technology, Heilongjiang University, Harbin 150080, China; Department of Computer Science and Technology, Shantou University, Shantou 515063, China; School of Mathematical Science, Heilongjiang University, Harbin 150080, China; School of Information Science and Engineering, Yanshan University, Qinhuangdao 066004, China

## Abstract

**Motivation:**

The microbes in human body play a crucial role in influencing the functions of drugs, as they can regulate the activities and toxicities of drugs. Most recent methods for predicting drug–microbe associations are based on graph learning. However, the relationships among multiple drugs and microbes are complex, diverse, and heterogeneous. Existing methods often fail to fully model the relationships. In addition, the attributes of drug–microbe pairs exhibit long-distance spatial correlations, which previous methods have not integrated effectively.

**Results:**

We propose a new prediction method named DHDMP which is designed to encode the relationships among multiple drugs and microbes and integrate the attributes of various neighbor nodes along with the pairwise long-distance correlations. First, we construct a hypergraph with dynamic topology, where each hyperedge represents a specific relationship among multiple drug nodes and microbe nodes. Considering the heterogeneity of node attributes across different categories, we developed a node category-sensitive hypergraph convolution network to encode these diverse relationships. Second, we construct homogeneous graphs for drugs and microbes respectively, as well as drug–microbe heterogeneous graph, facilitating the integration of features from both homogeneous and heterogeneous neighbors of each target node. Third, we introduce a graph convolutional network with cross-graph feature propagation ability to transfer node features from homogeneous to heterogeneous graphs for enhanced neighbor feature representation learning. The propagation strategy aids in the deep fusion of features from both types of neighbors. Finally, we design spatial cross-attention to encode the attributes of drug–microbe pairs, revealing long-distance correlations among multiple pairwise attribute patches. The comprehensive comparison experiments showed our method outperformed state-of-the-art methods for drug–microbe association prediction. The ablation studies demonstrated the effectiveness of node category-sensitive hypergraph convolution network, graph convolutional network with cross-graph feature propagation, and spatial cross-attention. Case studies on three drugs further showed DHDMP’s potential application in discovering the reliable candidate microbes for the interested drugs.

**Availability and implementation:**

Source codes and supplementary materials are available at https://github.com/pingxuan-hlju/DHDMP.

## 1 Introduction

The human body hosts a diverse community of microbes, including bacteria, viruses, protists, archaea, and fungi (Gill *et al.* 2006, De Lorenzi-Tognon *et al.* 2023). These microbial communities are closely linked to human health (Aggarwal *et al.* 2022, VanEvery *et al.* 2023), with studies suggesting that they can promote human metabolism and enhance the immune system (Ventura *et al.* 2009, Sommer and Bäckhed 2013). However, disruptions in these communities can lead to diseases such as hypertension and diabetes (Wen *et al.* 2008, Xiong *et al.* 2021).

Microbes can also chemically modify drugs to enhance their efficacy (Zimmermann *et al.* 2019, Kumbhare *et al.* 2023). For example, Vich Vila *et al.* (2020) have shown that the human gut microbiota is involved in drug metabolism processes, and compounds present in these drugs can promote the growth of gut bacteria. Conversely, microbes can develop resistance to drugs, negatively impacting human health (Uddin *et al.* 2021, Li *et al.* 2023). Therefore, predicting drug–microbe associations is beneficial for understanding drug mechanisms of action and drug–microbe interaction mechanisms.

Recently, the computational methods were proposed for predicting the miRNA–disease associations (Li *et al.* 2021), drug-target interactions (Li *et al.* 2017, Song *et al.* 2024). Computational prediction methods also have proven to be effective in predicting candidate microbes associated with drugs and screening reliable candidates for subsequent biological experiments. Researchers have proposed several approaches for predicting drug–microbe associations. Methods such as GCNMDA (Long *et al.* 2020a), EGATMDA (Long *et al.* 2020b), and SCSMDA (Tian *et al.* 2023) use simple graph convolutional networks (Kipf and Welling 2017) to aggregate neighbor information without differentiating between neighbor types. GSAMDA (Tan *et al.* 2022) and MDASAE (Fan *et al.* 2023) use sparse autoencoders, while Graph2MDA (Deng *et al.* 2022) employs a variational graph autoencoder (Kipf and Welling 2016) to learn node representation in the drug–microbe heterogeneous graph. However, these methods do not capture the complex relationships among multiple drugs and microbes. GACNNMDA (Ma *et al.* 2023) uses a convolutional neural network to learn features of drug–microbe node pairs but ignores the spatial dependencies between attributes of node pairs.

We present DHDMP, a method driven by node category-sensitive hypergraph convolutional networks, graph convolutional network with cross-graph feature propagation (GCNFP), and spatial cross-attention (SCA) for predicting drug-related microbes. Our method fuses the characteristics of multiple drugs and microbes that are related, and integrates the heterogeneity of multiple types of nodes and the long-distance spatial correlations of drug–microbe node pairs. The contributions of this article are summarized as follows:

- A microbe might affect the functions of multiple drugs, and multiple microbes are usually involved into the process that a drug exert its function. Thus, there are complex relationships among multiple drugs and multiple microbes. The hyperedges are designed to denote these relationships from multiple perspectives, and their embeddings are learnable. The learnable strategy is helpful for forming the hypergraph topology with dynamic changes.
- Most previous methods based on hypergraph learning only integrated the hypergraph topology and the node attributes and they ignored the heterogeneity of feature distributions of multiple categories of nodes. We designed their category features for each category of nodes to discriminate the heterogeneity of drug and microbe nodes. A node category-sensitive hypergraph convolutional networks (NHCN) is presented to encode the dynamic hypergraph topology and the node heterogeneity.
- A homogeneous graph composed of drug nodes, a homogeneous graph composed of microbe node, and a heterogeneous graph composed of both drug and microbe nodes, are established to reflect the similarities among the nodes from the same category and the associations among the nodes from different categories. In these graphs, a target node has its homogeneous neighbors and heterogeneous neighbors which reveal its characteristics from diverse perspectives. A feature propagation strategy is also proposed to supplement the node features from homogeneous graph to the heterogeneous graph learning. A GCNFP was designed to fuse the attributes of these neighbors and their topologies.
- The attribute embedding of a pair of drug and microbe node was constructed and there are long-distance spatial correlations among some attributes. We proposed a SCA to separate the pairwise attributes into multiple patches and integrate the long-distance correlations among these patches. The experimental results showed that our method achieved higher prediction performance than the compared advanced methods.

## 2 Materials and methods

**Figure 1.**
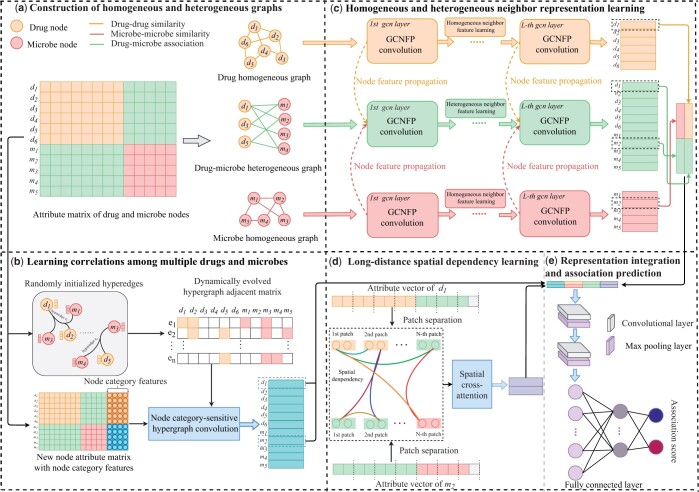
Overall framework of DHDMP model. (a) Construct the drug and microbe homogeneous graphs and drug–microbe heterogeneous graph. (b) Learn complex relationships among multiple drugs and microbes by NHCN. (c) Learn homogeneous and heterogeneous neighbor representations based on GCNFP. (d) Encode long-distance spatial correlations by SCA. (e) Integrate multiple representations and estimate the association score

We propose a new prediction model, DHDMP (as shown in Fig. 1), designed to predict the microbes associated with specific drugs. DHDMP consists of three components that respectively learn the complex relationships among multiple drugs and microbes, information from homogeneous and heterogeneous neighbors, and long-distance spatial correlations between pairs of drug and microbe nodes. We construct a hypergraph to represent the associations among multiple drugs and microbes and design a NHCN to capture the complex relationships among drugs and microbes. To integrate features from both homogeneous and heterogeneous neighbors of the target drug or microbe node, we propose a GCNFP. We introduce a SCA mechanism to capture long-distance spatial correlations between the features of drug and microbe node pairs. These components work together to enhance the predictive power of DHDMP in identifying drug–microbe associations.

### 2.1 Dataset

The associations between drugs and microbes, drug chemical structure similarity, Gaussian kernel similarity, and microbe sequence similarity were obtained from previous research (Deng *et al.* 2022). The dataset contains associations on 2470 pairs of drugs and microbes, covering 1373 drugs and 173 microbes. The data on drug–microbe associations were originally obtained from the Microbe–Drug Associations Database (MDAD) (Sun *et al.* 2018). Furthermore, 365 984 drug interactions were obtained from the DrugBank database (Wishart *et al.* 2018), while the raw microbe gene sequence data were retrieved from the National Center for Biotechnology Information (NCBI) database.

### 2.2 Construction of homogeneous and heterogeneous graph for drugs and microbes

We construct a drug homogeneous graph $G^{\text{drg}}=(V^{\text{drg}},E^{\text{drg}})$ and a microbe homogeneous graph $G^{\text{mic}}=(V^{\text{mic}},E^{\text{mic}})$. Here, $V^{\text{drg}}$ and $V^{\text{mic}}$ represent the sets of drug and microbe nodes, respectively. An edge $e_{i,j}^{\text{drg}}\in E^{\text{drg}}$ connects drug nodes $v_{i}^{\text{drg}},v_{j}^{\text{drg}}\in V^{\text{drg}}$, while microbe nodes $v_{i}^{\text{mic}},v_{j}^{\text{mic}}\in V^{\text{mic}}$ are connected by $e_{i,j}^{\text{mic}}\in E^{\text{mic}}$. Additionally, we construct a drug–microbe heterogeneous graph $G^{\text{drg-mic}}=(V^{\text{drg}}\cup V^{\text{mic}},E^{\text{drg-mic}})$, where an edge $e_{i,j}^{\text{drg-mic}}\in E^{\text{drg-mic}}$ connects a drug node $v_{i}^{\text{drg}}$ and a microbe node $v_{j}^{\text{mic}}$.

The drug similarity matrix $D^{\text{sim}}$ and microbial similarity matrix $M^{\text{sim}}$ are defined as follows,

(1) $$D^{\text{sim}}=(\frac{d_{i,j}^{\text{struct}}+d_{i,j}^{\text{gauss}}}{2})\in R^{N_{d}\times N_{d}},\text{if}\;v_{i},v_{j}\in V^{\text{drg}},$$

(2) $$M^{\text{sim}}=(m_{i,j}^{\text{sim}})\in R^{N_{m}\times N_{m}},\text{if}\;v_{i},v_{j}\in V^{\text{mic}},$$

where $N_{d}$ and $N_{m}$ represent the number of drugs and microbes, respectively. For a pair of drug nodes $v_{i}^{\text{drg}}$ and $v_{j}^{\text{drg}}$, their structural similarity $d_{i,j}^{\text{struct}}\in [0,1]$ is calculated by the SIMCOMP2 (Hattori *et al.* 2010) measurement based on their chemical structure graphs. The Gaussian kernel similarity between the *i-*th drug and the *j-*th one $d_{i,j}^{\text{gauss}}\in [0,1]$ is computed based on the two groups of microbes interacted with them,

(3) $$d_{i,j}^{\text{gauss}}=\text{exp}(-\mu ||A(v_{i}^{\text{drg}})-A(v_{j}^{\text{drg}})|{|}^{2}),$$

where $A(v_{i}^{\text{drg}})$ is the *i*th row of the drug–microbe association matrix. μ represents the normalized kernel bandwidth,

(4) $$\mu =\mu^{\prime }/\left(\frac{1}{N_{d}}\sum_{i=1}^{N_{d}}||A(v_{i}^{\text{drg}})|{|}^{2}\right),$$

where $\mu^{\prime }$ is the original bandwidth and it was set to 1. The final drug similarity is obtained by averaging the structural similarity and the Gaussian kernel similarity. Principal component analysis (PCA) (Weber *et al.* 2016) is used to calculate the similarity $m_{i,j}^{\text{sim}}\in [0,1]$ between microbe nodes $v_{i}^{\text{mic}}$ and $v_{j}^{\text{mic}}$ based on their gene sequences. These calculation methods were proposed by Long *et al.* (2020a). Since each row of the drug similarity matrix and the microbe similarity matrix contains the associations of the respective drug or microbe with all drugs and microbes, we can regard these matrices as the adjacency matrices of the drug homogeneous graph and the microbe homogeneous graph, respectively, and rename them as $R^{\text{drg}}$ and $R^{\text{mic}}$.

The adjacency matrix of the drug–microbe heterogeneous graph is defined as,

(5) $$\begin{array}{c} \begin{array}{c} R^{\text{drg-mic}}=(r_{i,j}^{\text{drg-mic}})\in R^{\left(N_{d}+N_{m})\times (N_{d}+N_{m}\right)}\\ \text{if}\;v_{i}\in V^{\text{drg}},v_{j}\in V^{\text{mic}} \end{array} \end{array},$$

where $r_{i,j}^{\text{drg-mic}}=1$ denotes the known association between the drug node $v_{i}^{\text{drg}}$ and the microbe node $v_{j}^{\text{mic}}$, and $r_{i,j}^{\text{drg-mic}}=0$ otherwise. The node attribute embedding matrix $Z\in R^{\left(N_{d}+N_{m})\times (N_{d}+N_{m}\right)}$ includes the drug similarity matrix $D^{\text{sim}}$, the microbe similarity matrix $M^{\text{sim}}$, and the drug–microbe association matrix $R^{\text{drg-mic}}$,

(6) $$\begin{array}{c} Z=\left[\begin{array}{cc} D^{\text{sim}} & R^{\text{drg-mic}}\\ R^{{\text{drg-mic}}^{T}} & M^{\text{sim}} \end{array}\right] \end{array},$$

where $R^{{\text{drg-mic}}^{T}}$ represents the transpose matrix of $R^{\text{drg-mic}}$. If a pair of microbes and drugs are associated or similar with more common drugs and microbes, they are more likely to be associated. The ith row of matrix Z, denoted as $Z_{i}$, contains the similarities and associations between node $v_{i}\in V^{\text{drg}}\cup V^{\text{mic}}$ and all drugs and microbes. Therefore, $Z_{i}$ can be considered the attribute of the node.

### 2.3 Multiple drugs and microbes association learning based on NHCN

A microbe often participates in multiple drug function processes, and multiple microbes may simultaneously participate in the same drug function process, leading to complex relationships between multiple drugs and microbes. To capture such relationships, we establish multiple hyperedges and form a drug–microbe hypergraph. We construct a hyperedge embedding matrix $E\in R^{(N_{d}+N_{m})\times N_{e}}$, where E is randomly initialized and trainable during the training process, and $N_{e}$ is the number of hyperedges. $e_{i,j}^{h}\in E$ indicates the tendency of the i-th node to belong to the j-th hyperedge. Combining the node attribute embedding matrix Z, we form a hypergraph with dynamically changing topology, where its adjacency matrix is denoted as H,

(7) H=Z·E.

The hypergraph topology is continuously updated during the training process, which is helpful for better reflecting the complex relationships among multiple drugs and microbes.

**Figure 2.**
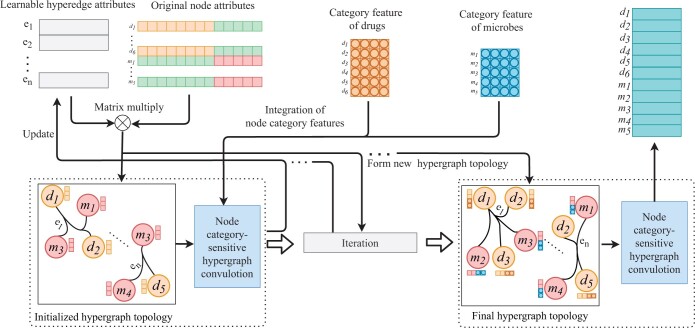
Illustration of forming and updating the dynamic hypergraph topology

In the hypergraph, there are two types of nodes: drugs and microbes, and their feature distribution is heterogeneous. We design category features to represent node types. The category feature matrix for all drug nodes is denoted as $Y_{d}\in R^{N_{d}\times \text{dim}}$, and for microbe nodes, it is denoted as $Y_{m}\in R^{N_{m}\times \text{dim}}$, where dim represents the dimension of the node category features. $Y_{d},Y_{m}$ are randomly initialized, and the category features of nodes with the same type have the same initial vector. $Y_{d},Y_{m}$ are stacked vertically to form the category feature matrix $Y\in R^{(N_{d}+N_{m})\times \text{dim}}$ for all drug and microbe nodes. The node attribute matrix embedded with category features is referred to as $X\in R^{\left(N_{d}+N_{m})\times (N_{d}+N_{m}+\text{dim}\right)}$. To encode the complex relationships among multiple drugs and microbes, we design NHCN (node category-sensitive hypergraph convolution network). After l layer encoding, we obtain the feature map ${\tilde{X}}^{l}$,

(8) $$\begin{array}{c} \begin{array}{c} {\tilde{X}}^{l}=\text{LeakyRe}LU\left(D_{B}^{-\frac{1}{2}}\cdot H\cdot D_{E}^{-1}\cdot H^{T}\cdot D_{B}^{-\frac{1}{2}}\cdot {\tilde{X}}^{l-1}\right)\\ l=1,2,\ldots ,L_{\text{hnn}} \end{array} \end{array},$$

where LeakyReLU(·) is the activation function, $L_{\text{hnn}}$ is the number of convolution layers of the hypergraph. $D_{B}$ and $D_{E}$ are the degree matrices of nodes and hyperedges, corresponding respectively to the sum of rows and columns in the hypergraph adjacency matrix. ${\tilde{X}}^{0}=X$ is an attribute matrix embedded with category features. We perform hypergraph convolution on all nodes and then update the hyperedge embedding matrix. The updated hyperedge embedding matrix is multiplied with the node attributes to form a new hypergraph topology. We perform hypergraph convolution on the new hypergraph topology and repeat the iteration process (as shown in Fig. 2). After $L_{\text{hnn}}$ rounds of hypergraph convolution, we obtain multi-node association representations ${\tilde{X}}_{\text{drg},i}^{L_{\text{hnn}}}$ and ${\tilde{X}}_{\text{mic},j}^{L_{\text{hnn}}}$ of $v_{i}^{\text{drg}}$ and $v_{j}^{\text{mic}}$, respectively. ${\tilde{X}}_{\text{drg},i}^{L_{\text{hnn}}}$ and ${\tilde{X}}_{\text{mic},j}^{L_{\text{hnn}}}$ are stacked to form the multi-node association representation for drug and microbe node pairs,

(9) $$\begin{array}{c} X_{i,j}^{\text{mna}}=\left[\begin{array}{c} {\tilde{X}}_{\text{drg},i}^{L_{\text{hnn}}}\\ {\tilde{X}}_{\text{mic},j}^{L_{\text{hnn}}} \end{array}\right] \end{array}.$$

### 2.4 Homogeneous and heterogeneous neighbor representation learning based on GCNFP

Neighboring nodes of a target node can reflect the characteristics of that node, we aggregate the features of neighboring nodes to form new features for the target node. A target node has both neighbors of the same type (homogeneous neighbors) and neighbors of different type (heterogeneous neighbors), each offering unique perspectives on the characteristics of the target node. The homogeneous neighbors of the target drug (or microbe) are nodes that have similarities, which from drug (or microbe) homogeneous graph. The heterogeneous neighbors associated with the target drug (or microbe) come from the drug–microbe heterogeneous graph, which reflects relationships between nodes of different types. Thus, the homogeneous neighbors reflect the characteristics of the target node from the perspective of similarity, while the heterogeneous neighbors reveal the characteristics of the target node from the perspective of association. To capture the distinct features reflected by homogeneous and heterogeneous neighbors of the target node, we design the GCNFP. First, we normalize the adjacency matrix $R^{\text{drg-mic}}$ of the drug–microbe heterogeneous graph to obtain ${\tilde{R}}^{\text{drg-mic}}$ (Kipf and Welling 2017),

(10) $${\tilde{R}}^{\text{drg-mic}}=D_{\text{drg-mic}}^{-\frac{1}{2}}\cdot R^{\text{drg-mic}}\cdot D_{\text{drg-mic}}^{-\frac{1}{2}},$$

here, $D_{\text{drg-mic}}^{-\frac{1}{2}}\cdot R^{\text{drg-mic}}\cdot D_{\text{drg-mic}}^{-\frac{1}{2}}$ represents the Laplacian normalization of $R^{\text{drg-mic}}$, where $D_{\text{drg-mic}}$ is the diagonal matrix and ${\left(D_{\text{drg-mic}}\right)}_{i,i}$ represents the number of non-zero entries in the row vector of the adjacency matrix $R^{\text{drg-mic}}$. Similarly, we compute normalized adjacency matrices ${\tilde{R}}^{\text{mic}}$ and ${\tilde{R}}^{\text{drg}}$ for the microbe and drug homogeneous graphs, respectively. We define the drug–microbe homogeneous adjacency matrix $A_{\text{homo}}\in R^{\left(N_{d}+N_{m})\times (N_{d}+N_{m}\right)}$ and the drug–microbe heterogeneous adjacency matrix $A_{\text{hete}}\in R^{\left(N_{d}+N_{m})\times (N_{d}+N_{m}\right)}$ as follows,

(11) $$\begin{array}{c} A_{\text{homo}}=\left[\begin{array}{cc} {\tilde{R}}^{\text{drg}} & O\\ O & {\tilde{R}}^{\text{mic}} \end{array}\right]A_{\text{hete}}=\left[\begin{array}{cc} O & {\tilde{R}}^{\text{drg-mic}}\\ {\tilde{R}}^{{\text{drg-mic}}^{T}} & O \end{array}\right] \end{array},$$

where O is a matrix with all elements being 0. After the l-th graph convolutional encoding layer, we can obtain the l-th order homogeneous neighbor representations $B_{\text{homo}}^{l}$ for all drugs and microbes,

(12) $$B_{\text{homo}}^{l}=A_{\text{homo}}\cdot B_{\text{homo}}^{l-1}\cdot W_{\text{homo}}^{l},l=1,2,\ldots ,L_{\text{gcn}},$$

where $B_{\text{homo}}^{0}=Z$ represents the initial node attribute matrix, $W_{\text{homo}}$ denotes the parameter matrix, and $L_{\text{gcn}}$ is the total number of layers in the GCNFP. Following $L_{\text{gcn}}$ graph convolutional iterations, we can derive first to $L_{\text{gcn}}$-th order homogeneous neighbor representations for both drugs and microbes. Given that these representations from homogeneous neighbors contain similarity information of drugs and microbes, while those from heterogeneous neighbors only encode association information between drugs and microbes, we devised a node feature propagation strategy (NFPS). This strategy leverages the information from homogeneous neighbors as auxiliary data to enhance the learning process of features from heterogeneous neighbors. Consequently, the l-th layer of heterogeneous neighbor representations $B_{\text{hete}}^{l}$ is expressed as,

(13) $$\begin{array}{c} B_{\text{hete}}^{l}=A_{\text{hete}}\cdot (B_{\text{hete}}^{l-1}\;||\;\alpha_{G}\cdot B_{\text{homo}}^{l})\cdot W_{\text{hete}}^{l},\\ l=1,2,\ldots ,L_{\text{gcn}}, \end{array}$$

where || denotes the concatenation operation, and $W_{\text{hete}}$ signifies the parameter matrix of the GCNFP. As each drug (microbe) node has its own unique information, we set a learnable attention coefficient for each node to capture the unique information. The attention coefficient vector of all the nodes is $\alpha_{g}\in R^{(N_{d}+N_{m})\times 1}$ and it is randomly initialized. The operation diag() may convert the vector $\alpha_{g}$ into a diagonal matrix $\alpha_{G}=\text{diag}(\alpha_{g})$. $B_{\text{hete}}^{0}=Z$ denotes the node attribute matrix of 0th layer, containing detailed information about each node. To construct a comprehensive homogeneous neighbor representation for $v_{j}^{\text{drg}}$ and $v_{j}^{\text{mic}}$, we concatenate the homogeneous neighbor representations from the 0-th to the $L_{\text{gcn}}$-th order. This yields multi-order homogeneous neighbor representations denoted as $B_{\text{homo},i}^{\text{drg}}(B_{\text{homo},j}^{\text{mic}})$,

(14) $$\begin{array}{c} \begin{array}{c} B_{\text{homo},i}^{\text{drg}}=\left[B_{\text{homo},i}^{0},\ldots ,B_{\text{homo},i}^{L_{\text{gcn}}}\right]\\ B_{\text{homo},j}^{\text{mic}}=\left[B_{\text{homo},j}^{0},\ldots ,B_{\text{homo},j}^{L_{\text{gcn}}}\right]. \end{array} \end{array}$$

We utilize a fully connected layer to transform the high-dimensional homogeneous neighbor representations $B_{\text{homo},i}^{\text{drg}}(B_{\text{homo},j}^{\text{mic}})$, which contain multi-order neighbor information, into a low-dimensional feature space. This transformation yields ${\tilde{B}}_{\text{homo},i}^{\text{drg}}({\tilde{B}}_{\text{homo},j}^{\text{mic}})$ as follows,

(15) $$\begin{array}{c} \begin{array}{c} {\tilde{B}}_{\text{homo},i}^{\text{drg}}=\text{Tan}\;h(W_{d}\cdot B_{\text{homo},i}^{\text{drg}}+b_{d})\\ {\tilde{B}}_{\text{homo},j}^{\text{mic}}=\text{Tan}\;h(W_{m}\cdot B_{\text{homo},j}^{\text{mic}}+b_{m}), \end{array} \end{array}$$

where $W_{d}$ and $W_{m}$ represent learnable parameter matrices, while $b_{d}$ and $b_{m}$ are bias vectors. Similar to formulas (14) and (15), we derive heterogeneous neighbor representations ${\tilde{B}}_{\text{hete},i}^{\text{drg}}$ and ${\tilde{B}}_{\text{hete},j}^{\text{mic}}$ for nodes $v_{i}^{\text{drg}}$ and $v_{j}^{\text{mic}}$. We combine these representations with the homogeneous neighbor representations ${\tilde{B}}_{\text{homo},i}^{\text{drg}}$ and ${\tilde{B}}_{\text{homo},j}^{\text{mic}}$ to obtain the homogeneous and heterogeneous representations of nodes $v_{i}^{\text{drg}}$ and $v_{j}^{\text{mic}}$, denoted as $B_{i,j}^{\text{hhn}}$,

(16) $$\begin{array}{c} B_{i,j}^{\text{hhn}}=\left[\begin{array}{cc} {\tilde{B}}_{\text{homo},i}^{\text{drg}} & {\tilde{B}}_{\text{hete},i}^{\text{drg}}\\ {\tilde{B}}_{\text{homo},j}^{\text{mic}} & {\tilde{B}}_{\text{hete},j}^{\text{mic}} \end{array}\right] \end{array}.$$

### 2.5 Long-distance spatial representation learning based on SCA

**Figure 3.**
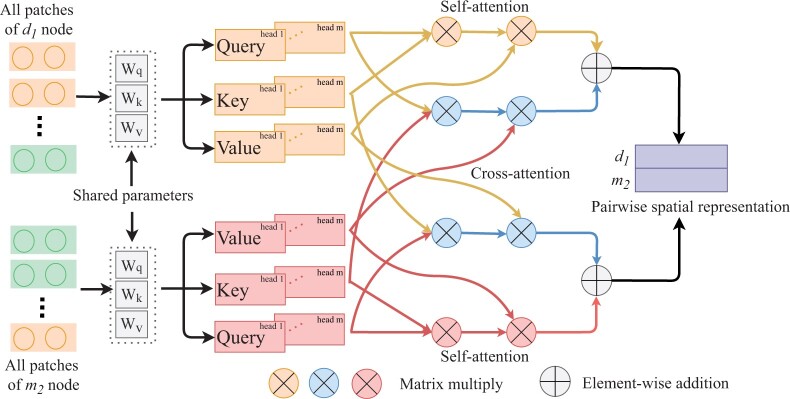
Encoding the long-distance correlations among multiple attribute patches of a pair of drug and microbe nodes

If $v_{i}^{\text{drg}}$ and $v_{j}^{\text{mic}}$ are similar or associated with more the same drugs and microbes, their attribute vectors exhibit more similarity in feature distribution. We reshape the attribute vectors $Z_{\text{drg},i}(Z_{\text{mic},j})\in R^{1\times (N_{d}+N_{m})}$ of $v_{i}^{\text{drg}}(v_{j}^{\text{mic}})$ into $Z_{\text{drg},i}^{\text{Pat}}(Z_{\text{mic},j}^{\text{Pat}})\in R^{N\times P}$, where P represents patch size and $N=\left(N_{d}+N_{m}\right)/P$. If $N_{d}+N_{m}$ is not divisible by P, we pad the end of $Z_{\text{drg},i}$ and $Z_{\text{mic},j}$ with zeros until $N_{d}+N_{m}$ is divisible by P. The reshaped matrices $Z_{\text{drg},i}^{\text{Pat}}$ and $Z_{\text{mic},j}^{\text{Pat}}$ are divided into N patches, where patches with similar features might be widely dispersed in space. The partitioning of attribute patches is helpful for designing an SCA mechanism to capture the spatial correlations among the patches (as shown in Fig. 3).

We introduce a multi-head attention mechanism (Dosovitskiy *et al.* 2020) to calculate attention from different perspectives to reduce bias in the learning process. Taking the m-th head also the last head attention as an example, we employ distinct linear transformations to produce drug query matrices $Q_{\text{drg},i}^{m}\in R^{N\times N_{\text{dim}}}$, key matrices $K_{\text{drg},i}^{m}\in R^{N\times N_{\text{dim}}}$, and value matrices $V_{\text{drg},i}^{m}\in R^{N\times N_{\text{dim}}}$ for each patch. These transformations are defined as follows,

(17) $$\begin{array}{c} \begin{array}{c} Q_{\text{drg},i}^{m}=Z_{\text{drg},i}^{\text{Pat}}\cdot W_{Q}^{m}\\ K_{\text{drg},i}^{m}=Z_{\text{drg},i}^{\text{Pat}}\cdot W_{K}^{m}\\ V_{\text{drg},i}^{m}=Z_{\text{drg},i}^{\text{Pat}}\cdot W_{V}^{m}, \end{array} \end{array}$$

where, $W_{Q}^{m},W_{K}^{m},W_{V}^{m}\in R^{P\times N_{\text{dim}}}$ are weight matrices. For all the patches of a pair of drug and microbe nodes, $W_{Q}^{m}$, $W_{K}^{m}$, $W_{V}^{m}$ are the shared weight matrices. It is beneficial for jointly capturing the spatial correlations among the features of the drug–microbe pair. For the N patches of $v_{i}^{\text{drg}}$ and $v_{j}^{\text{mic}}$, we calculate the spatial attention score matrix $Q_{\text{drg},i}^{m}K_{\text{drg},i}^{m^{T}}$, $Q_{\text{mic},j}^{m}K_{\text{mic},j}^{m^{T}}$, $Q_{\text{drg},i}^{m}K_{\text{mic},j}^{m^{T}}$, $Q_{\text{mic},j}^{m}K_{\text{drg},i}^{m^{T}}$ both within and between them. Then, we normalize $Q_{\text{drg},i}^{m}K_{\text{drg},i}^{m^{T}}(Q_{\text{drg},i}^{m}K_{\text{mic},j}^{m^{T}})$ and multiply it with $V_{\text{drg},i}^{m}(V_{\text{drg},j}^{m})$ to obtain the spatial correlations representation $F_{\text{drg},i}^{\text{self},m}(F_{\text{drg},i}^{\text{cros},m})$ for drugs from the perspective of self-attention (cross-attention),

(18) $$\begin{array}{c} \begin{array}{c} F_{\text{drg},i}^{\text{self},m}=\text{softmax}\left(\frac{Q_{\text{drg},i}^{m}K_{\text{drg},i}^{m^{T}}}{\sqrt{N_{\text{dim}}}}\right)V_{\text{drg},i}^{m}\\ F_{\text{drg},i}^{\text{cros},m}=\text{softmax}\left(\frac{Q_{\text{drg},i}^{m}K_{\text{mic},j}^{m^{T}}}{\sqrt{N_{\text{dim}}}}\right)V_{\text{mic},j}^{m}. \end{array} \end{array}$$

Similarly, we compute the spatial correlations representations $F_{\text{mic},j}^{\text{self},m}$ and $F_{\text{mic},j}^{\text{cros},m}$ for microbes under self-attention and cross-attention. The self-attention focuses on capturing the spatial correlations among the attributes of a drug (microbe) itself. The cross-attention concentrates on reveal the spatial correlations among the attributes of drug node and those of the microbe node. We combine the spatial correlations representations from these multiple perspectives to form $F_{\text{drg},i}^{m}$ and $F_{\text{mic},j}^{m}$ as follows,

(19) $$\begin{array}{c} \begin{array}{c} F_{\text{drg},i}^{m}=F_{\text{drg},i}^{\text{self},m}\;⊕\;F_{\text{drg},i}^{\text{cros},m}\\ F_{\text{mic},j}^{m}=F_{\text{mic},j}^{\text{self},m}\;⊕\;F_{\text{mic},j}^{\text{cros},m}, \end{array} \end{array}$$

where ⊕ represents element-wise addition. The final output is obtained by concatenating the results of all attention heads,

(20) $$\begin{array}{c} \begin{array}{c} F_{\text{drg},i}=[F_{\text{drg},i}^{1},\ldots ,F_{\text{drg},i}^{m}]\\ F_{\text{mic},j}=[F_{\text{mic},j}^{1},\ldots ,F_{\text{mic},j}^{m}]. \end{array} \end{array}$$

Subsequently, $F_{\text{drg},i}$ and $F_{\text{mic},j}$ are flattened as vectors, stacked vertically, and concatenated to represent the long-distance spatial correlations of node pairs $F_{i,j}^{\text{lds}}$,

(21) $$\begin{array}{c} F_{i,j}^{\text{lds}}=\left[\begin{array}{c} F_{\text{drg},i}\\ F_{\text{mic},j} \end{array}\right] \end{array}.$$

### 2.6 Multiple representations fusion and optimization

Multiple representations, including the multi-node association representation $X_{i,j}^{\text{mna}}$, homogeneous and heterogeneous neighbor representation $B_{i,j}^{\text{hhn}}$, and long-distance spatial correlations representation $F_{i,j}^{\text{lds}}$ of $v_{i}^{\text{drg}}$ and $v_{j}^{\text{mic}}$ are concatenated to obtain the final node pair representation $Z_{i,j}^{\text{fin}}$,

(22) $$Z_{i,j}^{\text{fin}}=\left[\begin{array}{ccc} X_{i,j}^{\text{mna}} & B_{i,j}^{\text{hhn}} & F_{i,j}^{\text{lds}} \end{array}\right].$$

We employ a convolution-pooling layer (as shown in Fig. 1e) to further integrate $Z_{i,j}^{\text{fin}}$. After the *l-*th convolution-pooling layer, the node pair feature is denoted as $Z_{i,j}^{\text{fin},l}$,

(23) $$Z_{i,j}^{\text{fin},l}=\text{max}\left(\sigma \left(W^{l}*Z_{i,j}^{\text{fin},l-1}+b^{l}\right)\right),l=1,2,$$

where $Z_{i,j}^{\text{fin},0}=Z_{i,j}^{\text{fin}}$ and σ(·) is activation function. max(·) denotes the max-pooling operation and * represents the convolution operation. $W^{l}$ and $b^{l}$ represent the weight matrix and bias vector of the *l-*th convolution-pooling layer, respectively. We name the feature obtained from the last convolution-pooling layer as ${\tilde{Z}}_{ij}^{\text{fin}}$, which enables the prediction of scores *p* associated with $v_{i}^{\text{drg}}$ and $v_{j}^{\text{mic}}$ through the fully connected layer,

(24) $$p=\text{softmax}\left(W_{\text{agg}}\cdot {\tilde{Z}}_{i,j}^{\text{fin}}+b_{\text{agg}}\right),$$

where $W_{\text{agg}}$ and $b_{\text{agg}}$ are the weight matrix and the bias vector, respectively. p[1](p[0]) represents the probability that drug node $v_{i}^{\text{drg}}$ has (does not have) an association with microbe node $v_{j}^{\text{mic}}$. The loss of our model is calculated based on the cross-entropy loss function,

(25) $$\text{loss}=-\sum_{i=1}^{N_{\text{bat}}}y[i]\;\text{log}(p[0])+(1-y[i])\;\text{log}(1-p[0]),$$

where $N_{\text{bat}}$ is the number of nodes per batch of training, and *y* represents the true association of a drug and a microbe.

## 3 Experimental evaluations and discussions

### 3.1 Parameter settings

We trained DHDMP using the Nvidia GeForce RTX 4060, utilizing the PyTorch framework and optimizing it with the Adam algorithm. The training parameters consisted of 80 epochs, a batch size of 32, and a learning rate of 0.0005. The numbers of the encoding layers in NHCN and GCNFP are selected from {1,2,3}. We conducted the experiments on all the combinations of layer number of NHCN and that of GCNFP (Supplementary Table S1). When NHCN and GCNFP contain two encoding layers, the model achieves the best prediction performance. Additionally, we established 32 hyperedges to capture complex relationships (Supplementary Table S2). Within the SCA mechanism, the patch size and feature dimension ($N_{\text{dim}}$) were set to 1×50, with eight attention heads utilized. The fusion of multiple representations involved two convolution layers with kernel sizes of 2×10 and 1×10, respectively, while the pooling layers utilized a window size of 1×10. We also studied the impact of datasets with different positive to negative ratio on DHDMP (Supplementary Table S3) and time complexity analysis are provided in Supplementary File S1.

### 3.2 Evaluation metrics

Five-fold cross-validation is used to evaluate the prediction performance of DHDMP and other comparative methods. All known drug–microbe associations are randomly divided into five equal parts, called positive samples. The number of unknown drug–microbe associations is much larger than the positive examples and these unknown associations are considered negative samples. During the training and testing process of each fold, four parts of positive examples and an equal number of randomly selected negative examples are used as the training set. The remaining positive and negative samples make up the test set.

Evaluation of the models is based on the area under the receiver operating characteristic curve (AUC) (Hajian-Tilaki 2013) and the area under the precision–recall curve (AUPR) (Saito and Rehmsmeier 2015). AUC and AUPR are computed for each fold, and the average of these 5-fold results yields the final AUC and AUPR scores. In addition, the top *k* recall rate of a microbe-related drug is also calculated, given the importance of identifying microbes associated with drugs for experimental purposes.

### 3.3 Ablation experiments

In our ablation experiments, we systematically remove components of our model—NHCN, GCNFP, and SCA—to assess their individual contributions to prediction performance (Table 1). When NHCN is excluded, we observe a decline in prediction performance by 2.7% and 13.2% in terms of AUC and AUPR compared to our final model. This highlights the crucial role of capturing the intricate relationships among multiple drugs and microbes in enhancing the accuracy of drug–microbe association prediction. Similarly, without the learning of homogeneous and heterogeneous neighbor representations by GCNFP leads to a decrease in AUC and AUPR by 1.5% and 13.0%, respectively, compared to the complete model. This confirms the significance of incorporating information from both homogeneous and heterogeneous neighbors, which contributes significantly to predicting drug–microbe associations. Furthermore, when SCA is not utilized, DHDMP experiences a reduction of 1.4% in AUC and 6.4% in AUPR. This demonstrates the effectiveness of SCA in capturing the long-distance spatial relationships between node pairs. After the category features were removed from the prediction model, its AUC and AUPR decreased by 1.7% and 5.5%, respectively. It indicated that the category features may reveal the heterogeneity of multiple kinds of nodes. After the node feature propagation was eliminated from the model, its AUC and AUPR decreased by 1.4% and 4.2%. It demonstrates that propagating node information from homogeneous graphs to heterogeneous graph facilitates heterogeneous neighbor feature learning. The results of our ablation experiments indicate that NHCN makes the most contribution to predicting drug–microbe associations. This is primarily attributed to the ability of the hypergraph to effectively represent the complex relationships among multiple drugs and microbes.

**Table 1.** Results of ablation studies of DHDMP.

NHCN	GCNFP	SCA	Category feature	NFPS	Average AUC	Average AUPR
✗	✓	✓	✗	✓	0.932	0.691
✓	✗	✓	✓	✗	0.944	0.693
✓	✓	✗	✓	✓	0.945	0.759
✓	✓	✓	✗	✓	0.942	0.768
✓	✓	✓	✓	✗	0.945	0.781
✓	✓	✓	✓	✓	**0.959**	**0.823**

The bold values are the highest ones.

### 3.4 Comparison with other methods

We assess our approach against six leading methods in the field: GCNMDA (Long *et al.* 2020a), EGATMDA (Long *et al.* 2020b), GSAMDA (Tan *et al.* 2022), GACNNMDA (Ma *et al.* 2023), SCSMDA (Tian *et al.* 2023), and NGMDA (Xuan *et al.* 2024). Each of these methods is trained using the optimal parameters mentioned in their original papers. To maintain consistency, we train our model and the comparative methods on the same dataset and use the same partitioning for training and testing. Below, you will find brief descriptions of these six comparative methods.

GCNMDA: This method constructs a network comprising drugs and microbes, leveraging their similarities and associations. By employing GCN and conditional random fields, the model predicts drugs relevant to microbes.

EGATMDA: This approach forms a heterogeneous network covering microbes, diseases, and drugs. It utilizes GCN with node-level attention and graph neural networks with graph-level attention to infer associations between microbes and drugs.

GSAMDA: In this model, drug and microbe similarities are computed using Gaussian interaction profiles (GIP) and Hamming interaction profiles (HIP). The features of drug and microbe nodes are learned through graph attention networks (GAT) and sparse autoencoders.

GACNNMDA: Similarity between drugs and microbes is determined based on GIP and HIP, and association scores between them are computed using convolutional neural networks.

SCSMDA: This method establishes an integrated network of drug and microbe similarities, constructing multiple meta-path-induced networks. It employs a framework based on graph convolution and graph contrastive learning to learn features of drug and microbe nodes.

NGMDA: It builds a drug–microbe heterogeneous graph and enhances node features through fully connected autoencoders. Additionally, a graph transformer is designed to infer associations between drugs and microbes.

Based on the results presented in Table 2, DHDMP achieved the highest average AUC at 95.9%, surpassing GCNMDA by 5.6%, EGATMDA by 1.9%, GACNNMDA by 11.6%, GSAMDA by 5.7%, SCSMDA by 4.3%, and NGMDA by 1.5%. Furthermore, DHDMP’s average AUPR stands at 82.3%, significantly outperforming GCNMDA, EGATMDA, GSAMDA, GACNNMDA, SCSMDA, and NGMDA by 50.8%, 51.6%, 57.6%, 62.7%, 48.3%, and 9.5%, respectively. Our method achieved the highest AUC and AUPR, with NGMDA demonstrating the second-best predictive performance. The superior performance of our method and NGMDA’s AUPR can be attributed largely to the incorporation and utilization of node-pair-level attributes. However, NGMDA falls short compared to our method due to its oversight of the intricate relationships between multiple drugs and microbes. GCNMDA, EGATMDA, and SCSMDA exhibit better performance compared to GSAMDA and GACNNMDA, possible reason is GSAMDA and GACNNMDA only learn node representations based on GATs or GCNs without considering the types of nodes and edges. GCNMDA, EGATMDA, and SCSMDA effectively learn the semantics of connections between drug and microbe meta-path nodes, thus yielding good performance.

**Table 2.** Average AUCs and AUPRs of our method and the compared methods over all the 1373 drugs.

Methods	AUC	AUPR
GCNMDA	0.903	0.315
EGATMDA	0.940	0.307
GSAMDA	0.902	0.247
GACNNMDA	0.843	0.196
SCSMDA	0.916	0.340
NGMDA	0.944	0.728
DHDMP	**0.959**	**0.823**

The bold values are the highest ones.

To determine whether our method’s AUC and AUPR are better than those of other methods, we conducted statistical tests. Using 5-fold cross-validation, we calculated the average AUCs and AUPRs for 1373 drugs for each method. Then, we performed Paired Wilcoxon tests to compare the AUCs and AUPRs of DHDMP with those of the other methods. Table 3 shows the statistical results, indicating a significant improvement (*P*-value <0.05).

**Table 3.** Results of the paired Wilcoxon test by comparing DHDMP and any of the other methods.

*P*-value	GCNMDA	EGATMDA	GACNNMDA	GSAMDA	SCSMDA	NGMDA
AUC	8.76e-159	5.82e-94	1.54e-165	1.28e-151	6.39e-164	3.47e-20
AUPR	5.25e-188	1.33e-162	1.78e-196	3.31e-190	1.18e-212	8.12e-33

**Figure 4.**
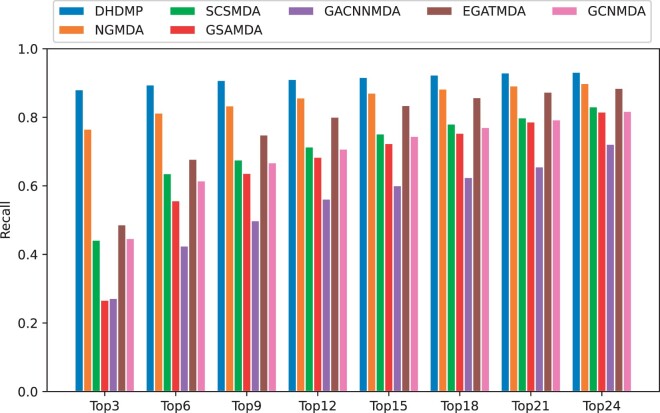
The average recall rates of drugs at multiple top *k* cutoffs

For each drug, we calculated the recall rates of candidate microbes at various top *k* values (Fig. 4). Our method consistently performs better than other methods for different *k* values. For example, when *k *=* *3, DHDMP achieves a recall rate of 88.1%, surpassing GCNMDA by 43.4%, EGATMDA by 38.9%, GACNNMDA by 60.9%, GSAMDA by 61.4%, SCSMDA by 43.9%, and NGMDA by 11.5%. In the range of k∈[6,9,12], DHDMP maintains the highest recall rate, while NGMDA closely follows with recall rates of 81.3%, 83.4%, and 85.7%, respectively. EGATMDA emerges as the third-best performing method with recall rates of 67.8%, 74.9%, and 80.1%. GCNMDA’s performance lags slightly behind SCSMDA, achieving recall rates of 61.5%, 66.8%, and 70.8%. While GSAMDA’s recall rates are slightly lower at 55.7%, 63.7%, and 68.4%, they still outperform GACNNMDA by 13.2%, 13.8%, and 12.1%, respectively.

### 3.5 Case studies

We conducted case studies on moxifloxacin, ciprofloxacin, and curcumin to demonstrate DHDMP’s ability to predict microbes that are relevant to these drugs. Moxifloxacin is commonly prescribed for upper and lower respiratory tract infections such as acute sinusitis, pneumonia, and skin and soft tissue infections. Ciprofloxacin is used to treat respiratory tract infections and sepsis, and as a preventive measure against infections in immunocompromised patients. Curcumin, a natural phenolic antioxidant, is known for its potential anti-cancer properties and its possible role in preventing Alzheimer’s disease. Our model was trained using all known associations and an equal number of randomly selected unknown microbe–drug associations. For each drug, the candidate microbes were ranked in descending order according to their association scores. The top 20 candidate microbes for each drug are regarded as its potential candidate microbes. We then analyzed the top 20 candidate microbes for moxifloxacin, ciprofloxacin, and curcumin. The detailed results can be found in Tables 4–6, respectively. The MDAD database provides clinically or experimentally supported associations between microbes and drugs, while the aBiofilm database compiles information on anti-biofilm agents relevant to over 140 microbes (Rajput *et al.* 2018). These two databases, along with relevant bioinformatics literature, were used to validate the predictions of drug–microbe associations. In the case of moxifloxacin, as listed in Table 4, MDAD contains four potential microbes, three of which are documented in aBiofilm, and evidence supporting 12 candidates can be found in the literature. Notably, studies (Dalhoff *et al.* 2020, Ontong *et al.* 2021) indicate that certain microbes such as *Escherichia coli*, *Streptococcus pneumoniae*, *Staphylococcus aureus*, and *Klebsiella pneumoniae* are inhibited by moxifloxacin, while *Listeria monocytogenes* and *Pseudomonas aeruginosa* exhibit resistance to it (Ince *et al.* 2003, Tahoun *et al.* 2017, Budzinskaya *et al.* 2019). Regarding ciprofloxacin, Table 5 reveals that nine of the potential microbes have recently been confirmed to have associations in the literature, 10 are recorded in MDAD, and seven can be found in aBiofilm. Similarly, the results presented in Table 6 for curcumin show that evidence from the aBiofilm and MDAD databases supports 18 of the potential microbes, with one confirmed in the literature.

**Table 4.** The top 20 candidate microbes related to moxifloxacin.

Rank	Microbe name	Evidence	Rank	Microbe name	Evidence
1	*Candida albicans*	aBiofilm, MDAD	11	*Salmonella enterica*	PMID: 22151215
2	*Haemophilus influenzae*	MDAD	12	*Vibrio campbellii*	Unconfirmed
3	*Stenotrophomonas maltophilia*	aBiofilm, MDAD	13	*Plasmodium falciparum*	PMID: 17214980
4	*Mycobacterium avium*	aBiofilm, MDAD	14	Influenza B virus	Unconfirmed
5	*Staphylococcus aureus*	PMID: 12654680	15	*Klebsiella pneumoniae*	PMID: 33406110
6	*Streptococcus mutans*	PMID: 29160117	16	Dihydropteroate synthase type-1	Unconfirmed
7	*Pseudomonas aeruginosa*	PMID: 31691651	17	*Streptococcus pneumoniae*	PMID: 31542319
8	*Escherichia coli*	PMID: 31542319	18	*Candida tropicalis*	Unconfirmed
9	*Listeria monocytogenes*	PMID: 28739228	19	Human immunodeficiency virus 1	PMID: 18441333
10	*Burkholderia pseudomallei*	PMID: 24502667	20	*Streptococcus sanguinis*	PMID: 10629010

**Table 5.** The top 20 candidate microbes related to ciprofloxacin.

Rank	Microbe name	Evidence	Rank	Microbe name	Evidence
1	*Bacillus subtilis*	MDAD	11	*Streptococcus mutans*	PMID: 30468214
2	*Staphylococcus aureus*	aBiofilm, MDAD	12	*Candida albicans*	PMID: 31471074
3	*Escherichia coli*	aBiofilm, MDAD	13	*Staphylococcus epidermidis*	PMID: 28481197
4	*Mycobacterium tuberculosis*	MDAD	14	*Listeria monocytogenes*	PMID: 28355096
5	*Proteus vulgaris*	aBiofilm, MDAD	15	*Enterococcus faecalis*	PMID: 27790716
6	*Haemophilus influenzae*	MDAD	16	*Klebsiella pneumoniae*	PMID: 27257956
7	*Providencia stuartii*	aBiofilm, MDAD	17	*Vibrio campbellii*	Unconfirmed
8	*Morganella morganii*	aBiofilm, MDAD	18	*Salmonella enterica*	PMID: 26933017
9	*Pseudomonas aeruginosa*	aBiofilm, MDAD	19	*Streptococcus pneumoniae*	PMID: 26100702
10	*Stenotrophomonas maltophilia*	aBiofilm, MDAD	20	*Vibrio harveyi*	PMID: 27247095

**Table 6.** The top 20 candidate microbes related to curcumin.

Rank	Microbe name	Evidence	Rank	Microbe name	Evidence
1	*Aeromonas hydrophila*	aBiofilm, MDAD	11	*Vibrio vulnificus*	aBiofilm, MDAD
2	*Citrobacter freundii*	aBiofilm, MDAD	12	*Raoultella ornithinolytica*	aBiofilm, MDAD
3	*Burkholderia cenocepacia*	aBiofilm, MDAD	13	*Staphylococcus aureus*	PMID: 32708619
4	*Candida albicans*	aBiofilm, MDAD	14	*Vibrio parahaemolyticus*	aBiofilm, MDAD
5	*Burkholderia multivorans*	aBiofilm, MDAD	15	*Pseudomonas japonica*	aBiofilm, MDAD
6	*Vibrio harveyi*	aBiofilm, MDAD	16	*Klebsiella variicola*	aBiofilm, MDAD
7	*Proteus mirabilis*	aBiofilm, MDAD	17	*Escherichia coli*	aBiofilm, MDAD
8	*Serratia marcescens*	aBiofilm, MDAD	18	*Streptococcus mutans*	aBiofilm, MDAD
9	*Enterobacter cancerogenus*	aBiofilm, MDAD	19	*Pseudomonas aeruginosa*	aBiofilm, MDAD
10	*Enterobacter ludwigi*	aBiofilm, MDAD	20	*Staphylococcus epidermidis*	Unconfirmed

### 3.6 Prediction of novel microbe–drug associations

We trained our model using all known drug–microbe associations and an equal number of randomly selected unknown associations. Subsequently, we applied our model to predict candidate microbes for 1373 drugs. The top 20 potential candidate microbes for each drug, as predicted by our model, are listed in Supplementary File S2.

## 4 Conclusion

We proposed a novel method to integrate the attributes of neighbor nodes in the homogeneous and heterogeneous graphs, and encode the long-distance dependencies within the pairwise attributes for predicting the drug-related microbes. The established hyperedges are beneficial for integrating the complex relationships among multiple drugs and microbes from various perspectives. The hypergraph topology was able to be formed dynamically according to the learnable hyperedge embeddings. The designed NHCNs deeply integrated the complex relationships and the heterogeneity of multi-category nodes. A graph convolutional network with node feature propagation enhanced the feature learning for the drug and microbe nodes by fusing the features from homogeneous and heterogeneous neighbors and their topologies. The presented SCA captured the long-distance spatial correlations among multiple attribute patches of the drug–microbe node pair. The cross-validation experimental results showed DHDMP’s higher AUC and AUPR than the compared six methods. The recall rates for the top-ranked candidates indicated that DHDMP was able to retrieve more actual drug–microbe associations. The case studies on three drugs showed DHDMP’s ability in screening the potential candidate microbes associated with an interested drug.

## Supplementary Material

btae562_Supplementary_Data
