# Supplementary material for: Dynamic category-sensitive hypergraph inferring and homo-heterogeneous neighbor feature learning for drug-related microbe prediction
Source: Bioinformatics. 2024 Sep 18;40(9):btae562. doi: 10.1093/bioinformatics/btae562 (PMC11441325; doi:10.1093/bioinformatics/btae562)
Supplement: btae562_Supplementary_Data [file btae562_supplementary_data.zip › Supplementary File SF1.pdf]

Supplementary Materials for “Dynamic category-sensitive hypergraph inferring and homo-heterogeneous neighbor feature learning for drug-related microbe prediction”

1 Parameter analysis

The numbers of the encoding layers in NHCN and GCNFP are selected from {1, 2, 3}. We conducted the experiments on all the combinations of layer number of NHCN and that of GCNFP. When NHCN and GCNFP contain two encoding layers, the model achieves the best prediction performance.

Supplementary Table ST1. Prediction results for the different layer numbers of NHCN and GCNFP.

Layer number of NHCN	Layer number of GCNFP	AUC	AUPR
1	1	0.947	0.774
1	2	0.942	0.815
1	3	0.943	0.788
2	1	0.948	0.811
2	2	0.959	0.823
2	3	0.946	0.806
3	1	0.945	0.796
3	2	0.945	0.812
3	3	0.947	0.783

The number of hyperedges is N_e. The value of N_e is selected from {16, 32, 64, 128}, and the model achieves the best performance (AUC=0.959 and AUPR=0.823) when N_e is 32. A smaller value of N_e might make it difficult for the prediction model to fully encode the biological characteristic of the associations among multiple drug and microbe nodes. On the other hand, a larger number might introduce the noisy data into the hypergraph learning.

Supplementary Table ST2. Prediction performance for the numbers of hyperedge.

number of hyperedges	AUC	AUPR
16	0.941	0.786
32	0.959	0.823
64	0.950	0.809
128	0.935	0.799

The ratio of the positive samples (the known drug-microbe associations) to the

negative samples (the unobserved drug-microbe associations) was nearly 1:95. When the ratio of the positive samples to the negative samples is 1:1, the prediction result was given in Table 2. We constructed the prediction models $DHDMP_{1:2}$, $DHDMP_{1:5}$, $DHDMP_{1:10}$ by utilizing the datasets with the positive and negative example ratios of 1:2, 1:5, and 1:10, respectively. The experimental results are shown in Supplementary Table ST3, and $DHDMP_{1:1}$ achieved the best prediction performance. The AUC and AUPR of $DHDMP_{1:2}$ decreased by 1.1% and 1.9% respectively compared to $DHDMP_{1:1}$. The AUC and AUPR of $DHDMP_{1:5}$ also decreased by 1.3% and 5.4% respectively. It indicated that utilizing more negative samples to train the prediction model caused the worse prediction performance.

Supplementary Table ST3. Effects of different ratios of positive samples to negative samples on the performance of the proposed model.

Ratios	1:1	1:2	1:5	1:10
AUC	0.959	0.948	0.946	0.931
AUPR	0.823	0.804	0.769	0.756

2 Time complexity analysis

DHDMP consists of NHCN, GCNFP, and SCA. NHCN contains 2 hypergraph convolutional encoding layers, GCNFP contains 2 graph convolutional encoding layers, and SCA calculates the long-distance spatial correlations of the pairwise attributes for all the node pairs. Thus, the time complexity is,

$$O((N_d + N_m)^2 \times (N_f + N_e + N_s)) + O(\sum_{i=1}^{N_d}\sum_{j=1}^{N_m} P^2 \times N_{dim})$$

where N_d is the number of drug nodes, N_m is the number of microbe nodes, and N_f is the attribute dimension with the category features, N_s is the attribute dimension without the category features, N_e is the number of hyperedges, P is the patch size, and N_{dim} is the feature dimension.
